# Minimal-invasive management of urological complications after kidney transplantation

**DOI:** 10.1007/s11255-021-02825-7

**Published:** 2021-03-02

**Authors:** Susanne Deininger, Silvio Nadalin, Bastian Amend, Martina Guthoff, Nils Heyne, Alfred Königsrainer, Jens Strohäker, Arnulf Stenzl, Steffen Rausch

**Affiliations:** 1 Department of Urology, University Hospital Tübingen, Eberhard Karls University, Hoppe-Seyler-Str. 3, 72076 Tübingen, Germany; 2 Department of Urology and Andrology, Salzburg University Hospital, Paracelsus Medical University, Salzburg, Austria; 3 Department of General and Transplant Surgery, University Hospital Tübingen, Eberhard Karls University, Tübingen, Germany; 4 Department of Internal Medicine IV, Section of Nephrology and Hypertension, University Hospital Tübingen, Eberhard Karls University, Tübingen, Germany

**Keywords:** Kidney transplantation, Complications, Endourology, Endoscopy, Minimal-invasive, Therapy

## Abstract

Kidney transplantation represents the gold standard treatment option for patients with end-stage renal disease. Improvements in surgical technique and pharmacologic treatment have continuously prolonged allograft survival in recent years. However, urological complications are frequently observed, leading to both postoperative morbidity and putative deterioration of allograft function. While open redo surgery in these patients is often accompanied by elevated surgical risk, endoscopic management of urological complications is an alternative, minimal-invasive option. In the present article, we reviewed the literature on relevant urological postoperative complications after kidney transplantation and describe preventive approaches during the pre-transplantation assessment and their management using minimal-invasive approaches.

## Introduction

Kidney transplantation (KT) is a therapeutic option for patients with end-stage renal disease, deliberating the recipients from the burden of regular dialysis. In 2018, over 90,000 KT were performed globally [1]. KT improves the survival of patients compared to peritoneal or hemo-dialysis [2]. Improvements in operative technique, donor and recipient selection, and immunosuppression have improved patients’ quality of life and also allograft survival in the last decades [3, 4]. From 2010 to 2014, the 1 and 5 years survival rate of deceased donor kidney allograft in the United States of America were 93.4 and 72.4%, respectively [5]. However, early and late-onset of surgical complications are frequently observed: Koçak et al. reported an overall complication rate of 15.7% in 362 cases of living donor KT, of which 8% were of urological nature (urinoma, ureteral stenosis, renal calculi, vesicoureteral reflux, lymphocele [6], ureteral necrosis [7]), while other surgical complications included vascular and wound healing problems, hematoma formation, and graft rupture [6]. Other authors reported on urological complication rates from 2.9 to 12.5% [7, 8].

The management of complications after KT may be challenging, due to an elevated risk after previous surgery and the need for ongoing immunosuppression in frequently comorbid patients [9]. Given recent advances in endoscopic urology, a minimal-invasive approach for urological complications might be a reasonable alternative. Moreover, meticulous preoperative urologic work-up prior to KT is demanded to identify risk profiles prior to surgery.

In the present article, we summarize available evidence for endoscopic and minimal-invasive treatment of post-KT urologic complications and provide putative approaches for clinical practice in adult patient. Given the paucity of high level evidence in the field, we conducted a targeted review of high-quality PubMed- indexed articles on the topic of urological complications in conjunction with KT. Moreover, we integrated additional aspects on the management of urological complications in the non-transplanted patient to further discuss the role of minimal-invasive approaches.

## Special features of pre-operative preparation and surgical techniques

### Pre-emptive urological assessment of the kidney transplant recipient and management of abnormal urinary tract

Pre-operative urological evaluation is an important prerequisite for preparation for KT and emphasis should be taken on the assessment of all functional and anatomical aspects of the urinary tract, as well as the exclusion of malignancy or chronic infection. Thorough patient history with voiding diary in presence of residual diuresis, physical examination, and ultrasound represent basic investigations which may be followed by uroflowmetry, cystoscopy, micturating cystourethrogram [10] and urodynamics [11], if indicated [12, 13]. Patients with a history of vesicoureteral reflux (VUR), pre-existing bladder emptying disorder, recurrent urinary tract infections (UTI), stone disease, urinary tract anomaly or after prior urologic surgery are at elevated risk to develop postoperative urologic complications [14] and may need extensive work-up. When the evaluation indicates bladder outlet obstruction (BOO) due to benign prostate hyperplasia (BPH) in male patients, the same stratification for either medical or surgical treatment according to non-transplanted patients may be performed [15]. Noteworthy, BPH was shown to be independently associated with urinary retention, urinary tract infection, and graft loss in patients after KT [16]. If surgical deobstruction e.g. with transurethral resection of the prostate (TUR-P) is indicated, it may either be performed prior [17] or after KT, although low or missing urine output before transplantation seems to promote bladder neck and urethral scarring [18]. In analogy, urethral strictures can be safely treated endoscopically with urethrotomy or open reconstruction surgery in dependence of the extent and the localization of the stricture [19, 20]. Using the endoscopic technique, it should be kept in mind that the risk of disease recurrence is elevated, compared to open urethral reconstruction. Regular urological follow-up in these patients is mandatory to screen for recurrent urinary retention with subsequent allograft damage.

Smaller surgical series have shown that transplantation in a dysfunctional bladder can be successful depending on the precautious selection of cases: high intravesical pressure (> 100 cmH_2_O peak pressure) and low bladder capacity (< 100 ml of volume) were identified to predispose to complications after KT [21]. In these cases, bladder augmentation may be discussed. In patients with a history of urethral valves [22], urinary diversion [23], or bladder augmentation [24] successful KT has been described but should be reserved for specialized centers.

In specific indications prior to transplantation, native nephrectomy may become necessary: unilateral or bilateral nephrectomy can improve pre-transplant clinical conditions of the patient in case of large proteinuria, UTI on the basis of VUR [25], and hypertension [26]. In cases of large polycystic kidneys, nephrectomy creates intraabdominal space and may remit possible symptoms like abdominal or flank pain, recurrent cyst infections or recurrent bleeding due to rupture of cysts [17].

### Technique of ureteral re-implantation and association to urological complications

The standard procedure for KT is an extraperitoneal approach after pelvic access, usually in the right, less common in the left iliac fossa (first described in 1951 from Kuss et al. [27]). Another surgical approach is a midline incision and placement of the graft intra-abdominally. This approach is chosen for example when the KT is combined with pancreas transplantation, in a complex vascular situation or after previous bilateral KT. The vessels are connected to the external or internal iliac artery on the one hand and to the external iliac vein on the other hand [28].

Subsequently, the ureteroneocystostomy (UCN) is conducted. Implantation techniques aim to achieve reflux protection while preserving optimal urine outlet and avoiding scar formation or inadequate perfusion of the transplant ureter. First anti-refluxive ureter implantation techniques have been developed in the 1950s by Politano imitating natural urinary tract conditions based on the assumption, that VUR can impair allograft function through increasing upper urinary tract pressure and the risk of pyelonephritis. Since then, the technique itself has been modified and other surgical techniques have been developed. The Politano–Leadbetter operating technique (PL) foresees an anterior cystostomy, the creation of a submucosal tunnel of 2–3 cm from inside the bladder and the feeding of the distal ureter through a new opening close to the trigonum [29, 30]. The Lich–Gregoir (LG) technique uses a 4 cm extravesical incision through the seromuscularis, and a 1 cm incision into the mucosa at the distal edge of the primary incision. The distal ureter is then sutured to the mucosa and the seromuscularis is closed over the ureter course to provide reflux protection [30]. Another extravesical approach (Woodruff) incises the seromuscularis from the outside of the bladder, conducts a smaller incision in the mucosa, implants the spatulated ureter into the mucosa but does not close the submucosal incision over the ureter course [31].

Thrasher et al. compared two subgroups of each 160 patients receiving ureter re-implantation by either the PL- technique or the LG- technique. The total number of urological complications was 9.4% in the PL- technique group and 3.7% in the LG technique group (*p* = 0.04). With 3.7%, the rate of uretero-vesical junction obstruction was significantly higher in the PL- technique group compared to the LG technique group (0.6%; p* = 0.05) [30]. A meta-analysis conducted by Alberts et al. in 2014 comparing the two main techniques arrives at the same result: the prevalence of both urinoma and hematuria were significantly reduced when using the LG- technique [32]. Today, anti-refluxive ureter implantation techniques are the gold standard in KT, and, based on the data mentioned above, guidelines strongly favor the use of the LG- approach. As an alternative, pyeloureteral or uretero-ureteral anastomosis can be discussed [33]. The perioperative placement of ureteral stents is a strongly recommended measure to avoid ureteral complications in the early postoperative course after KT [33]. In a study by Kumar et al., evaluating 670 living donor KTs, the application of ureteral stents reduced ureteral complications from 8.5 to 0.22% [34]. Nevertheless, stent-associated complications have also been reported: the incidence of UTI seems to be elevated in kidney graft recipients with ureteral stents [35], with the incidence increasing with the indwelling time of the stent [36].

## Endoscopic management of postoperative urologic complications in kidney allograft recipients

An overview of the data on endoscopic treatment of urological complications after KT can be found in Table 1.

**Table 1:** Minimal-invasive therapy options of urological complications after renal transplantation

Complication	Epidemiology in the KT collective	Minimal-invasive therapy option	Literature
US	Prevalence: 2–10% Manifestation: mostly within first 3 months after transplantation	Permanent supply with standard polymer ureteral stent	Success rate of 89.3% in benign US during a median FU of 16 months (*n* = 28; [38]) Success rate of 94% in intrinsic US during a FU of 3 months (*n* = 58; [39]) *Success rate of 96.9% in ureteral stent change with the assistance of a 14/16 French ureteral access sheath post KT (n* = *32; *[40]*)*
		Permanent supply with all-metal Resonance^®^ stent	Success rate of 80% in benign US during a median FU of 13 months (*n* = 20; [41]) Cost reduction of over 50% per year per patient in benign and malignant US (*n* = 50; [42])
		Permanent supply with self-expanding thermolabile Nitinol Memokath™ stent	*Success rate of 87% in US post KT during a mean FU of 4 years (n* = *8; *[43]*)*
		Endoscopic incision only (laser, cold knife or electrosurgical)	Success rate of 94.4% after ostium incision with a cold knife in the distal US after a FU of 3 and 24–36 months (*n* = 18; [50]) Success rate of 75% after benign US incision with cold knife after a minimum FU of 6 months (*n* = 18; [49]) No difference either in the complication or success rate after cold knife incision and LI of US (*n* = 50; [51])
		Endoscopic BD	Success rate of 57% in benign US treated with BD and 3 weeks of ureteral stent supply (*n* = 21; [54]) *Success rate of 12.5% in US post KT (n* = *8; *[57]*)* *Success rate of 53% in US post KT, mean time to recurrence 6.9 months (n* = *15; *[58]*)*
		Endoscopic BD plus LI	Success rate of 100% in pediatric patients with POM and US > 2 cm (*n* = 5; [59]) *Success rate of 67% in all US (n* = *6) and of 100% in US* < *1 cm (n* = *4) post KT during a mean FU of 52 months *[60]
		EAU guideline statement [33]	*Perform endoscopic treatment (BD or LI) for US* < *3 cm* *Perform surgical reconstruction in all US (*< *3 cm,* > *3 cm and late US)*
VUR	Prevalence: 10.5–86% Risk factors: deceased donor grafts [64], use of Lich- Gregoir ureteral re-implantation technique [62]	Injection of a bulking agent (DHAC) below the ureteral orifice/in the ureteral tunnel	No evidence of high grade (grade III-IV) VUR after the injection of DHAC in 100% in pediatric patients with febrile UTIs before treatment, no evidence of febrile UTI after treatment in 86.0% (*n* = 179; [73]) *Success rate of 78.9% after 2 injections of DHAC in patients with VUR post KT with* ≥ *3 UTIs/year (n* = *19), reduced incidence of UTIs from 4.89 to 1.31 (P* < *0.001) in a FU of 6 months *[74]
		EAU guideline statement [33]	*Perform endoscopic treatment as first line treatment for symptomatic VUR*
Urolithiasis	Prevalence: 1% Manifestation: after a median time of 28 months after KT x [77]	SWL	*Stone free rates of 100% after SWL in kidney stones post KT after a FU of 6 months (n* = *7; *[81]*)*
		RIRS/antegrade ureteroscopy	*Stone free rates of 91.7% after ante- (n* = *5) or retrograde (n* = *7) ureteroscopy in kidney stones post KT *[83] *Stone free rates of 71.4% after RIRS in kidney stones post KT (n* = *7), retrograde access impossible in n* = *2 *[84]
		PCNL	*Success of (mini-) PCNL in kidney stones post KT (n* = *1 each; *[85–87]*)*
		EAU guideline statement [33]	*Perform SWL or RIRS/antegrade ureteroscopy for stones* < *15 mm* *Perform PCNL for stones* > *20 mm*
Urinoma	Prevalence: 10% Manifestation: mostly within first 3 months after KT [90, 91]	Placement of nephrostomy, ante-/retrograde ureteral stenting Placement of transurethral catheter	
		Additional placement of drainage in larger fluid collections	[92]
LF	Prevalence: up to 20% [96–98]	Sonographic or radiological puncture and aspiration or drainage	*25% success rate in puncture and aspiration only (n* = *12) and 55% success rate in percutaneous drainage (n* = *18) for LF post KT *[102]
		Percutaneous fulguration of lymphocele via ureteroscope	*Treatment success and no symptoms after treatment of LF post KT after a FU of 6 months (n* = *1; *[103]*)*
		EAU guideline statement [33]	*Perform percutaneous drainage as first line treatment in large and symptomatic fistulas*

Italic indicates the data of kidney graft recipients

*US* ureteral stenosis, *BD* balloon dilatation, *DHAC* dextranomer/hyaluronic acid copolymer, *EAU* European Association of Urology, *FU* follow up, *KT* kidney transplantation, *LF* Lymphatic fistula, *LI* laser incision, *PCNL* percutaneous nephrolithotomy, *POM* primary obstructive megaureter, *RIRS* retrograde intrarenal surgery, *SWL* extracorporeal shock-wave lithotripsy, *VUR* vesicoureteral reflux, *UTI* urinary tract infection, *y/o* years old

### Ureteral stenosis

Ureteral stenosis (US) after KT may appear in form of ureterovesical junction obstruction (UVJO) and more proximal US. US is a complication that occurs in 2–10% of cases and typically becomes evident within the first 3 months after transplantation. Mostly, it is caused by ureteral ischemia due to loss of distal ureteral perfusion through graft explantation, less common causes are pre-existing anatomic anomaly, hematoma, lymphocele, calculi, tumor manifestation or scarring following UCN [37]. The diagnosis of obstruction may be challenging due to the possible absence of hydronephrosis. However, renal scintigraphy can help to reveal urinary obstruction. The European Association of Urology (EAU) guidelines recommend the placement of a nephrostomy as the primary approach and simultaneous diagnostic measure for UVJO [33, 34], followed by definite therapy where appropriate. Data on endoscopic treatment of US and UVJO after KT is available from a limited number of single-center series. Here, the principles of endoscopic treatment are adapted from clinical experience with the benign US in non-transplanted patients.

The permanent supply with standard polymer ureteral stent is a reasonable option in patients unfit or unwilling to undergo surgery: the literature reports of success rates of up to 100% in the intrinsic US [38, 39]. Stent exchange via the ureteral implantation site at the bladder dome can be challenging, so the use of assisting devices, like a 14/16 French ureteral access sheath, have been described [40].

To avoid the inconvenience and costs of regular stent exchanges, two different permanent metal stent approaches have been introduced. The all-metal Resonance^®^ stent has shown success rates of 80% in the benign US [41] while reducing material and operation costs over 50% per year per patient [42]. The self-expanding thermolabile nitinol stent Memokath™ has also been applied for the supply US after KT: In 2013, Bach et al. demonstrated a success rate of 87% with a medium indwelling time of the stent of 4 years and no perioperative complications [43].

Apart from the application of permanent stents, balloon dilation and US-incision are the two main operative principles described for endoscopic treatment of US. Here, the incision of the stricture may be performed by laser, cold knife or electrosurgical instrument. Usually, the incision is conducted in dorsolateral direction for the proximal US and in ventromedial direction for distal US [44]. The report success rates for the incision of distal US/UVJO varies between 62 and 88% [45–49]. In 2013, Arrabal-Martín published data of 18 patients with obstructive megaureter, obstruction secondary to bladder surgery, and orthotopic ureterocele with lithiasis with ureteral ostium incision. The incision was performed after placement of a guidewire or ureteral catheter via endoscopic scissors and cold cutting in medial and dorsal direction (5 o’clock position on the right side and 7 o’clock position on the left side). The procedure turned out to be a safe and effective treatment: all but one patient were treated on an outpatient basis, the grade of hydronephrosis decreased or disappeared after 3 months and the preoperative pain resolved completely in 17 out of 18 cases after 3 and 24–36 months [50].

In more proximal benign US, the endoscopic treatment also appeared to be a feasible therapeutic option with low complication rates: Lojanapiwat et al. report success rates of 75% after transurethral incision [49].

Comparing laser and cold knife incision in endoscopic US treatment, Dutkiewicz et al. in 2012 observed no differences in the success or complication rates [51]. Even though heterogeneous data exists as to which technique should be preferred [52], the current guidelines favor the antegrade approach and laser incision in short strictures [53].

Another endoscopic approach is the balloon dilation of the US: Byun et al. performed a 5–10 min balloon dilation of 21 benign US with a subsequent ureteral stent supply of 3 weeks releasing success rates of 57% [54].

**Fig. 1:**
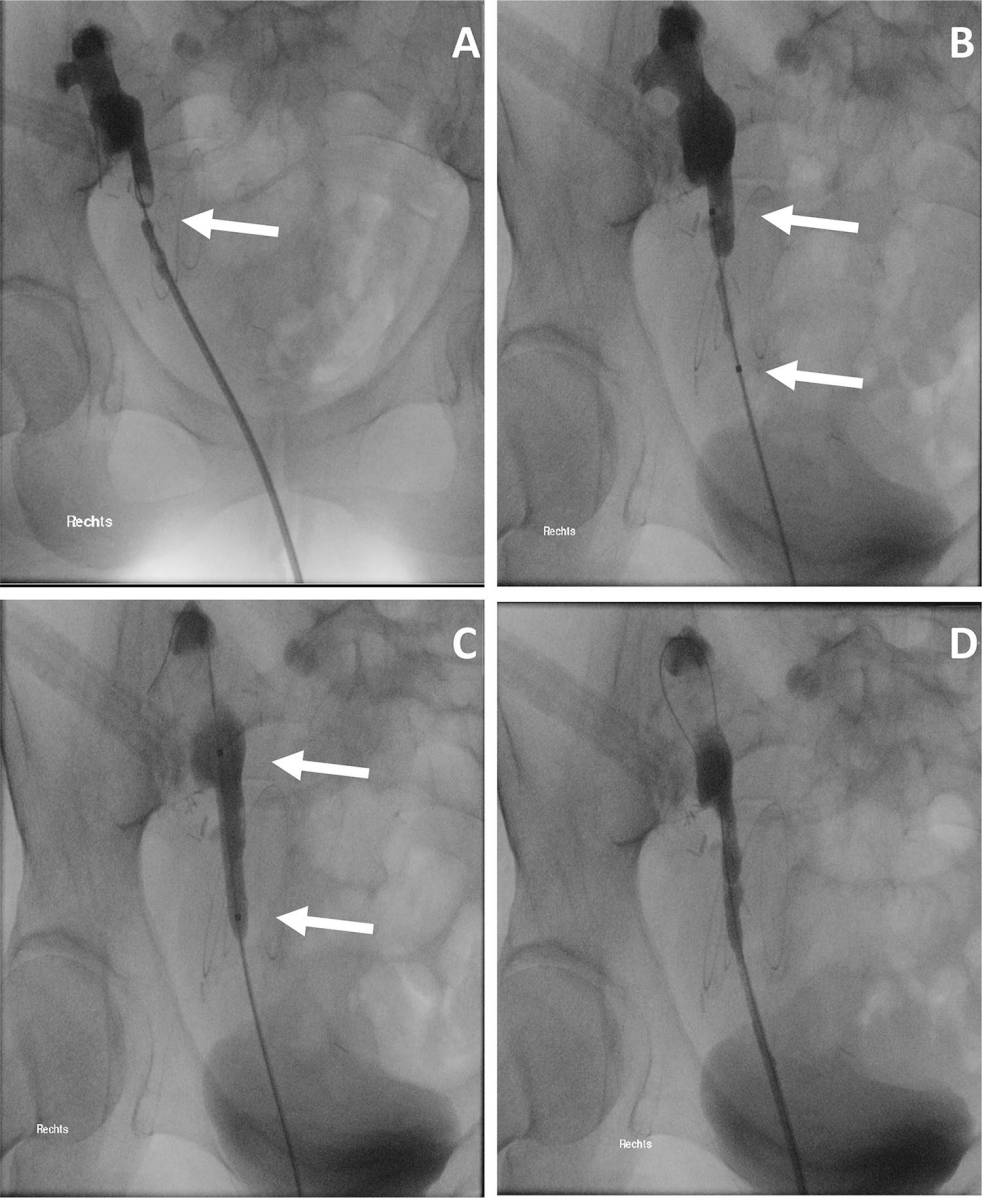
**a–d** Interventional balloon dilatation in secondary proximal ureteral stricture caused by superinfected lymphocele formation. **a** Radiographic illustration of short proximal ureter stricture (indicated by arrow). **b** Insertion of endoscopic balloon inflation device (arrow: radiopaque proximal and the distal end of the inflation balloon). **c** Activation of the dilation balloon and stricture dilation. **d** Final result with regular contrast passage after dilation

Balloon dilation seems efficient in benign, non-ischemic strictures shorter than 2 cm [54–56]. Figure 1 illustrates balloon dilatation in a proximal ureteral stenosis after KT. In the literature, balloon dilation of US in KT patients was reported with a success rate of only 12.5% after a median follow up of 11.4 months [57]. Gil-Sousa et al. described a US recurrence rate of 47% with a mean time to recurrence of 6.9 months after balloon dilation [58]. One reason for the unsatisfactory data might be the mostly ischemic etiology after KT, which warrants consideration in planning the procedure. The combination of both incision and dilation of the US seems to improve the success rate of balloon dilation alone. In 2012, Christman et al. described the endoscopic treatment in 17 pediatric patients with a median age of 7 years suffering from primary obstructive megaureter (POM) using combined laser and balloon dilatation: if shorter than 2 cm, the stricture was dilated with balloon only, if longer, the stricture was incised with laser and then dilated with a balloon. All patients experienced resolution of obstruction during the median follow-up time of 3.2 years [59]. In KT patients, Gdor et al. described a success rate of 67% in 6 KT patients with UVJO treated with holmium laser incision and balloon dilation at the same time, with the success rate being 100% in strictures with a maximum expansion of 1 cm in a mean follow up of 52 months [60].

While endoscopic techniques appear helpful in the treatment of US in KT patients and these interventions are reflected by contemporary guidelines, length of the stricture and etiology seem to be the most relevant predictive and selection parameters for the minimally invasive approach. Therefore, for US shorter than 3 cm, the panel recommends the endoscopic approach (balloon dilation or antegrade laser incision) or primary surgical reconstruction, for longer or recurrent US, surgical reconstruction is being favored [33, 61].

### Endoscopic management of vesicoureteral reflux (VUR) after KT

The incidence of vesicoureteral reflux (VUR) after KT varies from 10.5 [62] up to 86% [63] in literature, with data suggesting that deceased donor organs have a greater risk of developing VUR compared to living donor allografts [64]. Comparing the different UCN techniques, the incidence of VUR using the LG technique was reported to be higher than using the PL technique [62]. Even though older data indicates that VUR may increase the risk of long term graft failure [65], more recent data could not confirm the results: in a study cohort of 1008 adult kidney transplant recipients examined by Molenaar et al. in 2017, VUR did not have an impact on bacteriuria, allograft function after 3 and 12 months, as well as 1- and 5-year-graft survival [62]. Favi et al. showed similar results in 2017 [66]. Still, asymptomatic VUR needs to be distinguished from VUR with recurrent UTI. UTI is the most common type of infection following kidney transplantation (up to 47%) [67]. Especially in combination with VUR, UTI may manifest as graft pyelonephritis. A series of dimercaptosuccinic acid (DMSA) scans described that 69% of the pediatric patients suffering from VUR into the native kidneys and recurrent urinary tract infection showed renal scarring (*p* = 0.001) [68], a finding confirmed by pathological examinations [69]. In analogy, in high-grade VUR with recurrent pyelonephritis, therapeutic intervention may be indicated after KT.

While in pediatric patients with low-grade VUR, the disease tends to resolve spontaneously (13% annual rate for the first 5 years [70]), this can by nature not be expected after KT. However, conservative treatment with prophylactic antibiotics like trimethoprim (± sulfamethoxazole) and nitrofurantoin [71] can be administered in KT patients bearing in mind limitations of these compounds in patients with reduced GFR [72]. The injection of a bulking agent like dextranomer/hyaluronic acid copolymer (DHAC, Zuidex™) below the ureteral orifice or in the ureteral tunnel resolves the VUR by a single injection and only 3% of pediatric patients experience febrile UTI in the follow-up period of several years [73]. The injection of bulking agents in refluxive orifices of adult KT has also been described as a convenient therapeutic option: in 2011, Pichler et al. showed a significantly reduced incidence of UTI in 19 kidney transplant recipients with VUR after DHAC application in a median follow up time of 6.5 months [74]. Despite an overall low level of evidence in the KT population, current guidelines recommend the endoscopic approach as first-line treatment [33].

Apart from VUR in the kidney allograft, the presence of VUR in the native kidneys in case of reflux nephropathy increases the risk of UTI, with increasing numbers in higher grade reflux [75]. Techniques like native nephrectomy in high-grade reflux or distal ureter ligation in low-grade reflux during transplantation were shown to have a protective effect [76].

### Endoscopic management of urolithiasis in the transplanted patient

The formation of renal calculi is a rare complication after KT. The incidence of urolithiasis after KT is 1% after a median time of 28 months. The stone composition is merely calcium-based in 67%, struvite in 20% and uric acid in 13% [77]. Co-existing conditions of end-stage renal disease like hyperparathyroidism, hypercalciuria, hypophosphatemia, and UTI may increase the risk of calculi formation [78, 79]. Due to renal and ureteral denervation in kidney grafts, ureterolithiasis may occur without pain in transplanted patients causing other complications like hydronephrosis and acute kidney injury or hematuria. In the acute situation, ureteral stent placement resolves hydronephrosis. Like in native kidneys, the definite treatment depends on calculi genesis: in uric acid stones, the urine alkalisation with, for example, potassium sodium hydrogen citrate (Uralyt-U^®^) seems to be safe under strict potassium monitoring in patients with normal renal function [80]. In any other stone composition, extracorporeal shock-wave lithotripsy (SWL) is an option [81, 82]. The endoscopic treatment via ante- or retrograde flexible ureteroscopy has proven to be a safe and effective option although retrograde access can be challenging [83, 84]. For larger calculi, percutaneous nephrolithotomy (PCNL) can be performed via easy access because of the position of the allograft in the iliac fossa and the short puncture distance between skin and renal pelvis [85, 86]. Using mini- PCNL, the tissue damage to the graft can be reduced [87]. In 2018, Emiliani et al. reported that in 51 cases of urolithiasis therapies in transplanted kidneys including active surveillance, ante- and retrograde ureteroscopy, ESWL, PCNL and the open approach they found no loss of renal function through intervention and no case of allograft loss [88]. In the case of urolithiasis being present in a specimen at transplantation, an ex vivo ureteroscopy or pyelotomy should be performed to minimize the risk of post-transplant complication through spontaneous stone passing [89].

### Urinoma formation after KT

The formation of urinoma occurs in less than 10% of cases after KT mostly due to the insufficiency of the UCN, but also due to rupture of the bladder in case of long-term anuria before KT. It usually becomes evident in the early postoperative course of the first 90 days after surgery [90, 91]. The symptoms of urinoma vary from pain and infection to creeping creatinine and acute kidney injury. The placement of nephrostomy and antegrade ureteral stenting represent the first-line therapy in the acute phase as the retrograde approach is challenging and often challenging due to the implantation site of the ureter. In case of large fluid collections, additional drainage can be placed using ultrasound- or CT- guidance [92]. Moreover, the supply with transurethral catheter lowers pressure and minimizes reflux via the ureteral stent and thus supports recovery. The conservative regimen is often successful and the ureteral stent can usually be removed after 6–12 weeks [93, 94].

In case of a non-spontaneously resolving leak, extended fluid collection or ischemic ureter, a surgical revision is required. Distal ureteral injuries can be treated with open ureteral re-implantation. If the damaged ureteral part is extended, psoas-hitch technique may be combined with Boari tubularized bladder flap. An atrophic bladder in the recipient can make the formation of a bladder flap impossible, so uretero-ureterostomy to the native ureter or uretero-ileal interposition may be options in selected cases [95].

### Lymphatic fistula

The formation of lymphatic fistula after RT is a common complication occurring in up to 20% of the cases [96–98], caused by dissection of lymphatic vessels of the donor kidney during preparation and recipient pelvis during allograft implantation. Data indicates that the use of an absorbable polysaccharide hemostatic powder (HaemoCer™) can reduce the incidence of lymphatic fistula [99]. Only a minimum of patients requires interventional therapy in case of symptoms like pain or allograft dysfunction due to lymphocele infection or vascular compression. However, patients with lymphatic fistula in need of an intervention are at a higher overall risk of allograft rejection or delayed graft function [100]. Risk factors for lymphatic fistula formation include surgical preparation technique, implantation site, choice of immunosuppression (e.g. sirolimus/MMF/prednisolone), and recipient properties [101]. Available therapeutic options are puncture and aspiration, percutaneous drainage placement with or without sclerotherapy, and open or laparoscopic fenestration [102]. Endoscopic treatment of lymphatic fistula via percutaneous puncture, dilatation of the channel, insertion of flexible ureteroscope, and fulguration of lymphocele wall has been described [103]. Given the success rates of the more conventional techniques, however, this approach should be restricted to specific indications.

### Recurrent UTI after kidney transplantation

UTI is the most common type of infection following KT (up to 47%) [67]. Additionally, many patients develop asymptomatic bacteriuria (ASB) early after KT. While most centers treat ASB within 1–3 months ager KT, there is increasing data that this may be unnecessary [104].

Particularly in the first month after transplantation, the risk of UTI seems to be elevated through ureteral stents [105]. Early stent removal reduced the risk of UTI without increasing the risk of urinary leakage and should be performed around 3 weeks after KT [36]. However, even after stent removal for UTI the risk for repeat UTIs remains higher in patients who already suffered from stent-associated UTI [35].

All patients with persistent or recurrent UTI after KT should undergo a thorough assessment for anatomic or functional abnormalities of the urinary tract as already delineated earlier.

Risk factors for UTI after KT are female gender, immunosuppression, history of acute rejection, cytomegalovirus infection, UVJO [106], re-transplantation [67], polycystic kidney disease [107], diabetes mellitus [108] VUR in the native kidneys. While in the acute situation and for primary recurrence, prophylactic antibiotics should be administered, care should be taken to screen for functional or anatomical allograft pathologies.

Despite missing high evidence evaluation of behavioral and non-antibiotic prophylaxis for recurrent UTI in KT, patients should be advised to apply conservative measures in accordance with general recommendations. An excretion minimum of > 2 l/day, measures of genital hygiene, urine pH 5.8–6.5 (e.g. with an intake of vitamin C or methionine), application of vaginal estrogen/lactobacillus, and intake of cranberry products (juice/tablets) should be considered. In cases of residual urine, intermittent self-catheterization may be recommended. In addition, vaccination with inactivated species of *E. coli*, *Morganella morganii*, *Proteus*, *Klebsiella*, *Enterococcus faecalis* is an option [109].

The Pneumocystis jirovecii prophylaxis advised by many centers (3–6 months trimethoprim-sulfamethoxazole) seems to decrease the incidence of recurrent UTIs likewise [110].

## Conclusions

Urologic complications after kidney transplantation can lead to severe consequences up to chronic allograft dysfunction and eventually allograft loss. Successful transplantation is therefore heavily reliant on both thorough urologic work-up prior to transplantation, and early recognition of complications after surgery. Despite the lack of major systematic prospective evaluation, endoscopic treatment options offer minimally invasive options for patients with a variety of urological complications after kidney transplantation.
